# Simultaneous phosphorus recovery as vivianite crystallization and hydrogen generation from acidified oil wastewater by Fe-C micro-electrolysis

**DOI:** 10.3389/fchem.2026.1866760

**Published:** 2026-06-15

**Authors:** Kai Cui, Guangyu Xu, Hong Zhang, Jinpeng Yu, Fei Ma, Kun Guo

**Affiliations:** 1 School of Chemical Engineering and Technology, Xi’an Jiaotong University, Xi’an, China; 2 Shenmu Fuyou Energy Technology Co., Ltd, Shenmu, Shanxi, China

**Keywords:** acidified oil wastewater, Fe-C micro-electrolysis, hydrogen generation, phosphorus recovery, vivianite crystallization

## Abstract

Phosphorus recovery from industrial wastewater is an important route for alleviating phosphorus resource depletion and reducing eutrophication risk. Acidified oil wastewater generated during vegetable oil refining is characterized by strong acidity, high organic load, and high phosphorus content, making conventional treatment both costly and chemically intensive. Herein, an Fe-C micro-electrolysis process was developed to achieve phosphorus recovery from acidified oil wastewater through *in situ* vivianite crystallization, while simultaneously generating hydrogen as a value-added by-product. Commercial Fe-C filler was directly introduced into the strongly acidic wastewater, and the electrochemical reactions promoted the release of Fe^2+^, the consumption of protons, and the gradual increase in pH value, thereby creating favorable conditions for vivianite formation without the addition of exogenous iron salts or alkaline reagents. The effects of Fe-C filler particle size on phosphorus recovery efficiency, H_2_ generation, vivianite crystallization rate, as well as the removal efficiencies of chemical oxygen demand (COD) and NH_4_^+^-N were systematically investigated. Under the optimal condition using 3–6 mm Fe-C filler, the pH increased to 5.6 after 96 h of reaction, the phosphorus recovery efficiency reached 84.7%, and the vivianite crystallization rate reached 82.3%. Concurrently, per liter of wastewater treated, the process yielded 291 mL of H_2_ and approximately 100 g of vivianite, while simultaneously removing 48.2% of COD and 23.2% of NH_4_^+^-N. Compared with traditional chemical crystallization, this process realizes synergistic phosphorus recovery, hydrogen generation and pollutant degradation in a single system, providing a promising strategy for sustainable treatment and resource recovery from acidic phosphorus-rich industrial wastewater.

## Graphical Abstract

**Figure:**
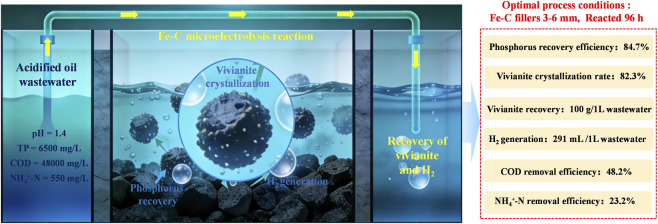

## 1 Introduction

Phosphorus (P) is an indispensable macronutrient for all living organisms and also a strategic non-renewable resource underpinning agricultural production, food supply chains and modern industrial development worldwide (Baker et al., 2024; Das et al., 2025). However, the global reserves of phosphate rock are finite and geographically unevenly distributed, while a substantial amount of phosphorus is incessantly lost into aquatic ecosystems through anthropogenic wastewater discharge (Slowinski et al., 2023). Excessive phosphorus input into aquatic systems is recognized as one of the primary drivers of water eutrophication, which triggers harmful algal blooms, dissolved oxygen depletion and progressive ecological degradation of aquatic habitats (Nesse et al., 2021). Consequently, phosphorus recovery from wastewater is no longer merely regarded as a conventional pollution control measure, but has evolved into an essential and integral component of circular resource management strategies for achieving sustainable phosphorus cycling (Gao et al., 2024; Mari Selvam et al., 2025).

Acidified oil wastewater is a typical industrial effluent generated during the chemical refining of vegetable oils. A large volume of such wastewater, rich in phosphorus-containing compounds, sulfates, residual oil, organic acids and dissolved organic matter, is produced in the acidification and washing steps of the refining process (Abd Nasir et al., 2019). This wastewater is typically characterized by an extremely low pH value (pH < 3.0–4.0), high chemical oxygen demand (COD) (40,000–60000 mg/L), elevated sulfate concentration (>6,000 mg/L) and high phosphorus content (Hamzah et al., 2019; Jiang et al., 2022). Owing to its complex composition and strong acidity, acidified oil wastewater cannot be effectively treated by conventional biological and physicochemical processes alone; direct neutralization of this wastewater (Jamaly et al., 2015). On the other hand, it consumes substantial amounts of alkaline reagents and generates secondary saline waste (Carrillo et al., 2020). In most existing treatment practices, the focus remains on the removal of COD and oil, while phosphorus in the wastewater, as a recoverable resource, is often overlooked (Adetunji and Olaniran, 2021; Yu et al., 2026).

Currently, mainstream technologies for phosphorus recovery from acidic industrial wastewater mainly include chemical precipitation, adsorption, biological treatment and membrane separation, each with distinctive merits and inherent limitations (Du et al., 2023; Huang et al., 2015; Nadagouda et al., 2024). Chemical precipitation, typified by struvite crystallization and exogenous ferrous iron-induced vivianite precipitation, achieves high phosphorus removal efficiency via rapid chemical dosing (Li et al., 2026). However, this technology relies heavily on the external addition of iron salts, magnesium sources and alkaline regulators, which leads to high reagent costs and stringent requirements for pH control, and is also prone to forming low-purity amorphous precipitates that impede subsequent resource utilization (Wu et al., 2019). Adsorption technology features mild operating conditions and high selectivity, yet its low adsorption capacity, cumbersome adsorbent desorption and regeneration (Sousa et al., 2025), as well as high material costs, render it unsuitable for the large-scale treatment of high-concentration wastewater (Worku et al., 2025). Biological phosphorus removal processes are environmentally friendly and cost-effective, but they are extremely sensitive to strongly acidic environments, resulting in poor microbial activity and unstable treatment efficiency when applied to acidified oil wastewater (Stokholm-Bjerregaard et al., 2017). Membrane separation technology enables efficient phosphorus separation and recovery, but it is plagued by membrane fouling, high capital and operational costs, and poor adaptability to complex wastewater matrices (Cheng et al., 2025).

Iron-carbon (Fe-C) micro-electrolysis, based on galvanic cell corrosion, is widely studied for refractory wastewater treatment owing to simple operation, low cost and strong adaptability to extreme water quality (Duan et al., 2022; Lin et al., 2019; Zhang et al., 2018). Preliminary studies confirm it facilitates in-situ Fe^2+^ release and spontaneous pH elevation via proton consumption (Guo et al., 2025), creating favorable thermodynamic conditions for vivianite crystallization and enabling reagent-free P recovery from acidic wastewater (Zhang et al., 2024). However, existing research focuses on neutral/weakly acidic wastewater, with scarce systematic investigations on ultra-strongly acidic acidified oil wastewater (Wang L. J. et al., 2023). Additionally, studies on the simultaneous generation of hydrogen as a clean energy carrier during the iron-carbon micro-electrolysis process are rarely reported. In particular, the quantitative relationships among hydrogen production, phosphorus recovery efficiency, and pollutant removal rate remain unclear, which severely hinders its engineering application and functional expansion in the treatment of acidic P-rich wastewater.

Against this background, this study innovatively developed a Fe-C micro-electrolysis process without the addition of exogenous chemical reagents (e.g., iron salts and alkaline reagents), which recovers phosphorus from acidified oil wastewater through in-situ formation of vivianite crystals while producing high-value by-product hydrogen. The effects of different Fe-C filler particle sizes on the pH of the reaction system, Fe^2+^ release, phosphorus recovery efficiency, COD and ammonia nitrogen removal efficiencies, as well as hydrogen generation were systematically investigated, and the optimal operating parameters for efficient phosphorus recovery from acidified oil wastewater in the form of vivianite and simultaneous hydrogen production were determined. It is worth noting that the target wastewater in this study is acidified oil-containing wastewater, which is significantly different from sludge digester liquor (neutral pH, low organic load, and low sulfate content) and livestock wastewater (neutral to weakly alkaline pH, low phosphorus concentration). The acidified oil-containing wastewater is characterized by extremely strong acidity, high-COD, high sulfate concentration, and high phosphorus content (Hamzah et al., 2019). The complex and harsh matrix of this wastewater poses unique challenges to phosphorus recovery, which have not been addressed in previous studies on Fe-C micro-electrolysis. Thus, this study enriches the theoretical system of Fe-C micro-electrolysis for resource recovery from acidic industrial wastewater and offers a sustainable strategy for green treatment and circular utilization of acidified oil wastewater, with significant academic value and broad engineering prospects.

## 2 Materials and methods

### 2.1 Characteristics of acidified oil wastewater and Fe-C fillers

The acidified oil wastewater samples used in this study were collected from a vegetable oil processing plant located in Weifang City, Shandong Province, China. Its main physicochemical properties are as follows: highly acidic with a pH of 1.4 ± 0.2, total phosphorus (TP) content of 6,500 ± 100 mg/L, COD of 48,000 ± 500 mg/L, NH_4_^+^-N concentration of 550 ± 50 mg/L, and the concentrations of calcium ion (Ca^2+^) and magnesium ion (Mg^2+^) were 300 mg/L and 500 mg/L, respectively. The concentrations of other ions in the wastewater were extremely low and could be neglected in this study. All wastewater samples were immediately stored under refrigeration at 0 °C after collection to inhibit microbial activity and ensure the stability of subsequent experiments. Additionally, due to the presence of a small amount of impurities such as calcium scale precipitates in the collected raw wastewater, the wastewater samples need to be centrifuged at 7,500 rpm for 10 min in a low-temperature centrifuge at 4 °C prior to the experiment, and the supernatant is then collected for subsequent batch reaction experiments and analytical determinations.

The experiment adopted commercialized Fe-C fillers as the micro-electrolysis medium. The Fe-C micro-electrolysis filler used was the YC-TC type product manufactured by Shandong Yichuang Environmental Protection Technology Co., Ltd. The main physical and compositional parameters of this filler are presented as follows (provided by the supplier): specific surface area was 1.5 m^2^/g, porosity ≥65%, the mass fraction of catalyst (calculated as metal oxides such as Fe^3+^) was 5%–7%, the mass fraction of activator (mainly inorganic acid salts) was 3%, bulk density was 1.3 t/m^3^, the mass fraction of iron element was 75% ± 5%, and the mass fraction of carbon element was 10%–15%. The particle size of Fe-C filler directly affects its specific surface area, reaction contact efficiency, and mass transfer rate, thereby altering the Fe^2+^ release amount and reaction interface interaction intensity in the system, and ultimately influencing the phosphorus precipitation recovery effect (e.g., vivianite formation efficiency) and hydrogen production capacity. Therefore, several typical particle size ranges (3–6 mm, 5–8 mm, and 8–12 mm) of fillers were selected for comparative experiments to determine the optimal particle size range of Fe-C filler for the treatment of acidified oil wastewater.

### 2.2 Experimental procedure of vivianite crystallization by Fe-C micro-electrolysis

**FIGURE 1:**
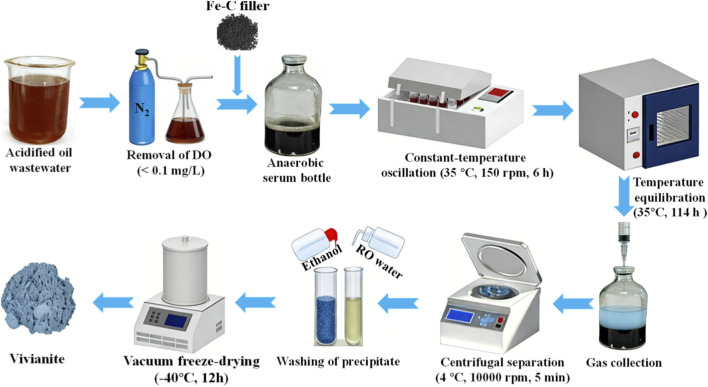
The process flow for vivianite crystallization from acidified oil wastewater by Fe-C micro-electrolysis.

The process flow for vivianite crystallization from acidified oil wastewater by Fe-C micro-electrolysis was illustrated in Figure 1. The specific operation is as follows: Firstly, high-purity nitrogen gas (N_2_ ≥ 99.99%) was continuously bubbled into the wastewater supernatant at a fixed flow rate of 50 mL/min for 30 min to thoroughly eliminate dissolved oxygen (DO), establishing a strictly anaerobic atmosphere with DO concentration below 0.1 mg/L, thereby effectively suppressing the oxidation of Fe^2+^. Subsequently, 80 mL of the deoxygenated supernatant was aseptically transferred into 125 mL serum bottles inside an anaerobic glove box, and pre-weighed Fe-C micro-electrolysis filler was added at a solid-liquid ratio of 1:10 (w/v). Each bottle was immediately sealed tightly with a butyl rubber stopper and aluminum crimp cap to guarantee airtightness of the reaction system.

The sealed serum bottles were then placed in a constant-temperature shaking incubator (Model: ZHWY-2102C) for batch reaction under controlled conditions: 35 °C, 150 r/min shaking for 2 h, followed by static settling for 4 h to facilitate crystal precipitation and phase separation. After the settling stage, the serum bottles were transferred to a constant-temperature incubator at 35 °C for 114 h of temperature equilibration to promote the uniform growth of vivianite crystalline particles. Headspace gas was collected using a gas-tight syringe equipped with a sealed sampling system, and the volume fraction of H_2_ was determined via gas chromatography; the cumulative H_2_ yield was further calculated in combination with the headspace gas volume.

Upon completion of each batch reaction, the mixed liquor was centrifuged at 10,000 r/min for 5 min at 4 °C, and the supernatant was collected for the determination of pH, Fe^2+^ concentration, COD, NH_4_^+^-N concentration, and TP concentration. The obtained precipitate was alternately washed three times with anhydrous ethanol (to reduce the loss of vivianite during the washing process and protect the integrity of its crystal structure) and oxygen-free ultrapure water (reverse osmosis (RO) water deoxygenated by nitrogen purging for ≥30 min). Finally, the washed precipitate was freeze-dried under vacuum at −40 °C for 12 h using a vacuum freeze dryer (Model: Labconco FreeZone 2.5) to obtain a vivianite-enriched precipitate, followed by the calculation of vivianite yield and mineralogical characterization.

### 2.3 Analytical methods

The determination methods for the physicochemical parameters of the wastewater samples collected from the reaction system are as follows: The solution pH was directly measured using a calibrated benchtop pH meter. TP content was determined via the molybdenum-antimony-ascorbic acid spectrophotometric method, while PO_4_^3-^-P concentration was quantified by the ascorbic acid reduction-molybdenum blue colorimetric method (Sun et al., 2024). COD was tested using the standard potassium dichromate oxidation method (Kolb et al., 2017), and NH_4_^+^-N concentration was detected via Nessler’s reagent spectrophotometry (Wu and Cao, 2013). Detailed operating procedures, instrumental parameter settings, and quality control protocols for all the above analytical methods are comprehensively described in references (Kolb et al., 2017; Sun et al., 2024; Wu and Cao, 2013).

Analytical method for Fe^2+^ concentration: an aliquot of 5 mL well-mixed reaction solution was transferred into an anaerobic centrifuge tube and centrifuged at 10,000 rpm for 5 min. The obtained supernatant was immediately vacuum-filtered using a 0.22 μm syringe filter within an anaerobic glovebox, and the collected filtrate was used for the quantitative determination of Fe^2+^ concentration. In this study, the concentrations of Fe^2+^ were measured and analyzed via inductively coupled plasma optical emission spectrometry (ICP-OES). The detailed experimental procedures and calculation protocols of this analytical method can be found in the previously reported literature (Cui et al., 2026; Liu et al., 2020).

Determination of H_2_ component: A 20 mL aliquot of the gas sample was introduced into a gas chromatograph (GC, Model 7890B, Agilent Technologies, USA) via a sampling injector for qualitative and quantitative analysis of gas compositions. The GC system was equipped with multiple complementary components, including a thermal conductivity detector (TCD), a flame ionization detector (FID), an HP-PLOT Al_2_O_3_ S PT column, a Porapak Q column, and a HyasSep Q + MolSieve 5 A column. Pure helium gas was employed as the carrier gas at a constant flow rate of 69 mL/min. The temperatures of the chromatographic column, injector, and detectors were maintained at 90 °C, 250 °C, and 250 °C, respectively (Yu et al., 2025).

Mineralogical characterization of vivianite-enriched precipitates: The mineral phase composition of the precipitates was identified by X-ray diffraction (XRD) (Paskin et al., 2025). The surface morphology and elemental composition were characterized via scanning electron microscopy coupled with energy-dispersive X-ray spectroscopy (SEM-EDS) (Tran et al., 2024). Furthermore, X-ray photoelectron spectroscopy (XPS) was employed to determine the crystallographic characteristics of the precipitates, including elemental chemical states, elemental distribution and crystal structure (Miyazawa et al., 2026). The detailed instrument models, testing parameters, data processing procedures and quality control protocols for all above characterizations are comprehensively documented in references (Miyazawa et al., 2026; Paskin et al., 2025; Tran et al., 2024).

### 2.4 Calculation methods

In this study, the Hupfer sequential extraction method was adopted to fractionate phosphorus from the freeze-dried vivianite crystals (Van Kuppevelt et al., 2025). The five dominant phosphorus speciation forms and detailed compositional information during the Hupfer extraction procedure are summarized in Supplementary Table S1. The calculation formulas for phosphorus recovery efficiency (Equation 1) and crystallization rate (Equation 2) are defined as follows:

$$Phosphorus\;recovery\;efficiency\;\left(\%\right)=\frac{{C_{pi}‐C}_{pe}}{C_{pi}}\times 100\%$$ (1)

$$Crystallization\;rate\;\left(\%\right)=\frac{C_{Fe‐P}\times V\times Mv}{2\times Mp\times mr}\times 100\%$$ (2)

Where *C*_*pi*_ and *C*_*pe*_ represent the phosphorus concentration in the raw wastewater and the solution after the reaction, respectively; *Mp* denotes the molar mass of phosphorus, *Mv* is the molar mass of vivianite, *V* is the volume of extractant, *mr* is the mass of the freeze-dried vivianite crystal used for extraction, and *C*_*Fe-P*_ is the concentration of Fe-P determined via the Hupfer method.

COD removal efficiency (Equation 3) was calculated as:

$$COD\;removal\;efficiency\;\left(\%\right)=\frac{{COD}_{i}\;\left(mg/L\right)-{COD}_{e}\;\left(mg/L\right)}{{COD}_{i}\;\left(mg/L\right)}\times 100\%$$ (3)

Where COD_*i*_ and COD_*e*_ represent the COD concentration in the raw wastewater and the solution after the reaction, respectively.

NH_4_^+^-N removal efficiency (Equation 4) was calculated as:

$${NH}_{4}^{+}-N\;removal\;efficiency\;\left(\%\right)=\frac{{\left({NH}_{4}^{+}-N\right)}_{i}\;\left(mg/L\right)-{\left({NH}_{4}^{+}-N\right)}_{e}\;\left(mg/L\right)}{{\left({NH}_{4}^{+}-N\right)}_{i}\;\left(mg/L\right)}\times 100\%$$ (4)

Where (NH_4_^+^-N)_*i*_ and (NH_4_^+^-N)_*e*_ represent the NH_4_^+^-N concentration in the raw wastewater and the solution after the reaction, respectively.

## 3 Result and discussion

### 3.1 Effect of Fe-C fillers particle size on pH variation

**FIGURE 2:**
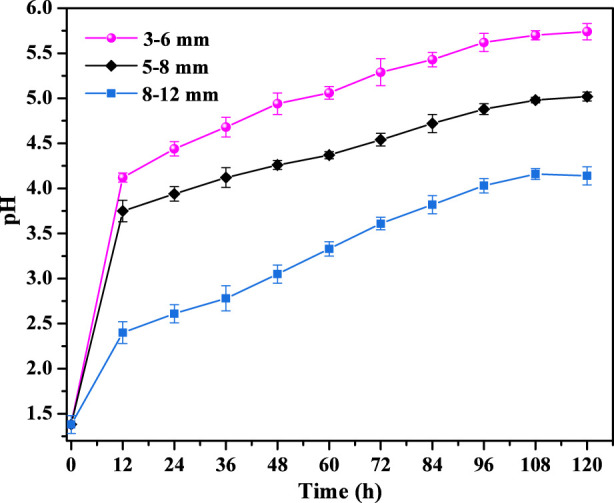
Effect of Fe-C filler particle size on the variation of pH in the reaction system.

The pH value is a pivotal control parameter governing the nucleation and precipitation of vivianite in acidic oil wastewater (Liu et al., 2018). Under strongly acidic conditions, a large amount of H^+^ in the system reacts with PO_4_^3-^ to form HPO_4_^2-^, H_2_PO_4_^−^, or H_3_PO_4_, which significantly reduces the concentration of free PO_4_^3-^ in the solution. Meanwhile, the activity of Fe^2+^ is also affected by the acidic environment. Consequently, the ion activity product of Fe^2+^ and PO_4_^3-^ is difficult to reach the solubility product (Ksp ≈1.0 × 10^−36^ at 25 °C) of vivianite, thereby inhibiting its direct nucleation and crystallization (Li and Sheng, 2021). As illustrated in Figure 2, the raw acidified oil wastewater exhibited strong acidity with an initial pH of 1.4. During the Fe-C micro-electrolysis process, the system pH increased continuously with prolonged reaction time, and the rising rate slowed down remarkably after 96 h. Specifically, the pH of the system with 3–6 mm Fe-C fillers increased to 5.77 at 120 h, while those of the 5–8 mm and 8–12 mm filler groups reached 5.02 and only 4.16, respectively. This finding is particularly significant because it indicates that the wastewater environment changed from a strongly acidic state to a pH (≥3.5) lower limit chemically favorable for vivianite formation without any external pH adjustment (Aguilar-Pozo et al., 2025). The gradual increase in pH rather than a sudden one also has benefits, as it may reduce the formation of competitive precipitates and facilitate the stabilization of Fe^2+^ for phosphate sequestration.

Notably, there is a significant negative correlation existed between the filler particle size and pH enhancement efficiency. The smaller particle sizes (3–6 mm) provided a larger specific surface area and shorter ion diffusion paths (Luo et al., 2026), which accelerated the rates of electron transfer and H^+^ consumption, endowing the 3–6 mm filler group with the optimal pH regulation capacity. Previous studies have shown that when the pH exceeded 3.5, the concentrations of Fe^2+^ and PO_4_^3-​^ in the solution could satisfy the threshold of the thermodynamic saturation index (SI) for vivianite precipitation (Supplementary Figure S1) (La Iglesia, 2009). Furthermore, within the pH range of 3.5–10, the SI value increased monotonically with increasing pH value. Consequently, the pH of all three filler groups exceeded 4.0 after 120 h of reaction, meeting the basic thermodynamic conditions for vivianite crystallization (Cui et al., 2024). Among these groups, the 3–6 mm Fe-C fillers, with their high-efficiency H^+^ consumption capacity and favorable reaction kinetics, were the most conducive to the directional formation of vivianite crystals.

### 3.2 Effect of Fe-C fillers particle size on Fe^2+^ release

**FIGURE 3:**
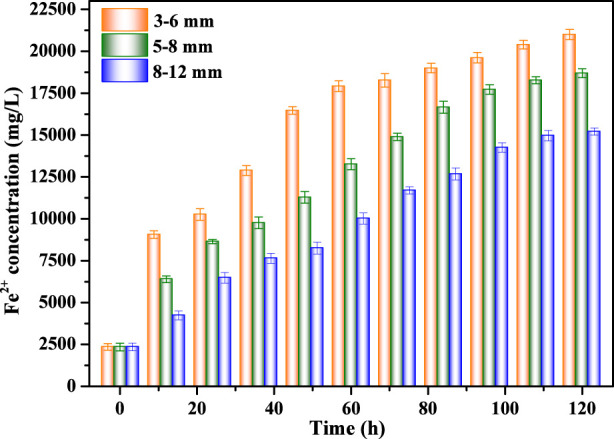
Effect of Fe-C filler particle size on Fe^2+^ concentration in the reaction system.

In the Fe-C micro-electrolysis system, the sustained release of Fe^2+^ constitutes another core chemical process driving the vivianite crystallization (Fan et al., 2025; Paskin et al., 2025). Metallic iron acts as a sacrificial anode in the micro-galvanic cells, continuously leaching Fe^2+^ during electrochemical corrosion. Therefore, the Fe^2+^ concentration measured in the liquid phase not only reflects the iron corrosion rate, but also serves as a comprehensive indicator of the dynamic competitive relationship between Fe^2+^ generation and consumption. In Figure 3, the Fe^2+^ concentration exhibited a continuous increasing trend with reaction time, confirming the stable operation of micro-galvanic cell reactions in the Fe-C system. Critically, the filler particle size exerted a significant regulatory effect on the Fe^2+^ accumulation efficiency. The smaller particle sizes (3–6 mm) provided a larger specific surface area per unit mass of filler, shorter electron transfer pathways and higher corrosion interface activity (Luo et al., 2026; Wang X. J. et al., 2023), thus resulting in a markedly higher Fe^2+^ release rate and final accumulated concentration compared with the larger particle size groups (5–8 mm and 8–12 mm). It should be noted that the initial Fe^2+^ concentration in the wastewater was much lower than the phosphorus content, failing to meet the stoichiometric ratio (Fe:P = 1.5:1) required for vivianite crystallization (Paskin et al., 2023; Paskin et al., 2025).

Theoretical calculations indicated that the favorable Fe^2+^ concentration corresponding to this molar ratio was 17,500 mg/L. Experimental data showed that the 3–6 mm filler group reached this threshold at 60 h, whereas the 5–8 mm filler group required 96 h. After 120 h of reaction, the Fe^2+^ concentrations in all filler groups stably exceeded 17,500 mg/L. Combined with the aforementioned pH enhancement results (all three filler groups achieved pH > 4.0), it can be confirmed that the Fe-C micro-electrolysis system enables the in-situ and directional crystallization of vivianite under the simultaneous satisfaction of dual constraints: thermodynamic conditions (pH > 3.5) and stoichiometric requirements (Fe:P ≥ 1.5:1) (Liu et al., 2018; Paskin et al., 2023). Moreover, it is worth noting that the effluent produced from the reaction contains extremely high dissolved Fe^2+^ (up to 20,000 mg/L) and residual COD (24,800 mg/L). Direct discharge without advanced treatment would pose considerable ecological risks to the aquatic environment. Therefore, a post-treatment unit is required for this process to effectively remove residual Fe^2+^ and COD, thereby ensuring that the effluent quality complies with the discharge limits for industrial wastewater (Yuan et al., 2024).

### 3.3 Effect of Fe-C fillers particle size on phosphorus recovery efficiency

**FIGURE 4:**
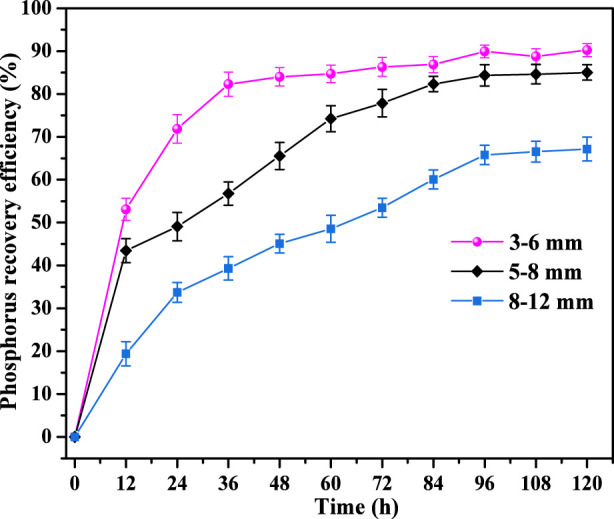
Effect of Fe-C filler particle size on phosphorus recovery efficiency in the reaction system.

Phosphorus recovery performance is the most critical indicator for evaluating the phosphorus resource utilization efficiency of the Fe-C micro-electrolysis process. The total phosphorus recovery efficiency of the system increased continuously with the extension of electrolysis time (Figure 4), which was mainly attributed to the synergistic effect of two aspects. Firstly, Fe-C fillers sustainedly released Fe^2+^ during the micro-electrolysis process (Figure 3), providing the metal cations required for vivianite crystallization *in situ*. Secondly, Ca^2+^ and Mg^2+^ coexisting in the wastewater could also react with phosphate to form insoluble precipitates such as calcium phosphate and magnesium phosphate (Banke et al., 2025), which constituted an auxiliary phosphorus removal pathway and thus improved the total phosphorus recovery efficiency of the system.

The Fe-C filler particle size exerted a significant regulatory effect on the total phosphorus recovery performance. Smaller particle sizes (3–6 mm) featured a larger specific surface area (Luo et al., 2026), a higher density of electrochemically active sites and a greater Fe^2+^ release flux. Furthermore, under the stirring/oscillation conditions of the reaction, interfacial micro-peeling was more likely to occur on the filler surface, exposing fresh reaction interfaces and thus further enhancing the mass transfer and reaction kinetics (Zhou et al., 2023). For these reasons, the 3–6 mm filler group exhibited the optimal phosphorus recovery performance. After 96 h of reaction, the system tended to reach a steady state, with the total phosphorus recovery efficiencies of the three filler groups (3–6 mm, 5–8 mm, and 8–12 mm) reaching 89.6%, 84.3%, and 65.7%, respectively.

In addition, a kinetic modeling analysis was conducted for the total phosphorus removal process in this study (Table 1). Based on classical reaction kinetic theory and extensive literature validation (Rousseau et al., 2004), the attenuation of total phosphorus concentration in this system conformed to the characteristics of the pseudo-first-order reaction kinetics, whose integral form is expressed as: *P*_*t*​_ = *P​e* ^*-kt*^. In this equation, P_0_​ and P_t_​ represent the initial total phosphorus concentration and that at time t (mg/L), respectively, and k is the apparent pseudo-first-order rate constant (min^-1^). Exponential regression was performed on the experimental data using nonlinear fitting software, and the optimal fitting equation was obtained as: *P*_*t​*_ = 6,276.7e^−0.00497t^ (R = 0.9684). This result further verifies the effectiveness of the phosphorus recovery process from acidified oily wastewater using Fe-C filler in this study.

**TABLE 1:** The variation of total phosphorus concentration and its corresponding kinetic parameters in the reaction system with the reaction time.

Time (h)	P_t_ (mg/L)	-ln (P_t_/P_0_)	-(1/P_0_-1/P_t)_
0	6,500	0.00000	0.00000
12	3,049	0.75694	0.00017
24	1830	1.267,624	0.00039
36	1,151	1.73161	0.00072
48	780	2.12026	0.00113
60	604	2.37623	0.00152
72	499	2.56525	0.00185
84	528	2.51084	0.00174
96	330	2.97986	0.00287
108	404	2.77741	0.002320
120	375	2.85249	0.002512

### 3.4 Effects of Fe-C fillers on the removal efficiency of COD and NH_4_^+^-N

**FIGURE 5:**
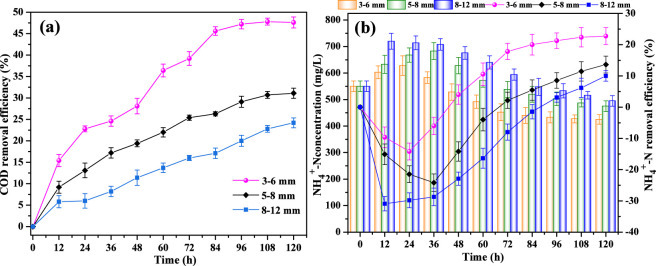
Effects of Fe-C filler particle size on the removal efficiency of COD **(a)** and NH_4_^+^-N **(b)** in the reaction system.

While achieving phosphorus resource recovery, the Fe-C micro-electrolysis process in this technology also exhibits a significant synergistic effect on the removal of organic pollutants in wastewater. The COD removal efficiency increased continuously with the extension of electrolysis time and tended to flatten after 96 h. At 120 h, the COD removal efficiency corresponding to three groups of fillers with different particle sizes were 48.2%, 31.1%, and 24.4%, respectively (Figure 5a). This regularity indicates that the COD removal efficiency is jointly regulated by electrolysis time and the specific surface area of the filler. On the one hand, the Fe-C system spontaneously forms micro-primary batteries in an acidic medium, Fe^0^ acts as the anode to undergo oxidative dissolution, while the carbon material acts as the cathode to promote electron transfer and interfacial reactions (Li et al., 2025). The generated Fe^2+^-mediated reaction system reduced some refractory organic compounds, thereby improving their chemical degradability (Wu et al., 2023). On the other hand, with the progress of the reaction, the pH of the system gradually increased, and Fe^2+^ and Fe^3+^ were sequentially hydrolyzed to form Fe (OH)_2_ and Fe (OH)_3_ colloids. The latter relying on its strong adsorption capacity and flocculation effect, further effectively removed organic compounds in the wastewater.

Additionally, the COD removal efficiency (48.2%) in the reaction system was relatively lower compared with that of similar Fe-C systems in this study. The possible reasons are as follows: On the one hand, the high organic load of the acidified oil wastewater in this study significantly increased the difficulty of degradation. On the other hand, the main components of COD in the acidified oil wastewater are refractory organic compounds (e.g., residual oil, long-chain fatty acids, and aromatic compounds), which are difficult to be degraded by Fe-C micro-electrolysis alone. The Fe^2+^/Fe^3+^ redox cycle in the Fe-C system mainly degrades readily degradable organic matter, while refractory organic compounds can only be partially degraded or adsorbed. In addition, the focus of this study is on phosphorus recovery and hydrogen production, and the reaction conditions were optimized for vivianite crystallization (e.g., 35 °C, anaerobic environment), which are not the optimal conditions for COD degradation. In Fe-C systems, the optimal temperature for COD degradation is typically 40 °C–50 °C, and aerobic conditions can promote the oxidation of organic matter (Chen et al., 2025). Therefore, in future work, Fe-C micro-electrolysis can be coupled with biological treatment processes to further improve the COD removal efficiency.

The Fe-C micro-electrolysis process also leads to the removal of part of ammonia nitrogen. Although NH_4_^+^-N recovery is not the primary goal of this system, this synergistic removal behavior is worthy of discussion because it further enhances the comprehensive treatment potential of the process. In Figure 5b, the NH_4_^+^-N concentration presents dynamic change trend of “first increasing and then decreasing”, which increases significantly in the initial stage of the reaction (0–12 h), then gradually decreases, and finally achieves a net removal rate of 10.0%–23.2% at 120 h (varying with filler particle size). This phenomenon originates from the temporal coupling of three parallel mechanisms: (i) Reductive transformation-dominated stage (0–12 h). The adsorbed hydrogen atoms precipitated on the cathode surface have strong reducibility, which can gradually reduce nitrogen-containing oxidized pollutants in wastewater to amino groups (Wang et al., 2025). Meanwhile, Fe^0^ can also directly reduce nitrate and nitrite under strong acidic conditions (pH < 4) (Cai et al., 2024), but this process decays rapidly as the pH of the system increases (Figure 2 shows that pH > 4 after 12 h). Therefore, the accumulation of ammonia nitrogen in the early stage is mainly attributed to these two reductive pathways; (ii) Contribution of biological/chemical hydrolysis. The residual proteins in wastewater hydrolyze under acidic conditions and Fe^2+^ catalysis, releasing amino acids which further undergo deamination, forming a secondary source of ammonia nitrogen increase; (iii) Synergistic solidification and removal stage (after 12 h). With the progress of the reaction, Fe^2+^ combines with OH^−^ to form Fe(OH)_2_ colloids, whose surface positive charges can electrostatically adsorb negatively charged ions such as NH_4_^+^ and HPO_4_^2-^, and form composite flocs with Fe(OH)_2_ as the core through co-precipitation, net-capturing and sweeping, and floc wrapping effects. Meanwhile, charged colloidal particles migrate to the filler surface under the drive of electric field and undergo electrophoretic deposition, further enhancing the interfacial enrichment and solid-phase interception of pollutants.

### 3.5 Effect of Fe-C fillers particle size on H_2_ generation

**FIGURE 6:**
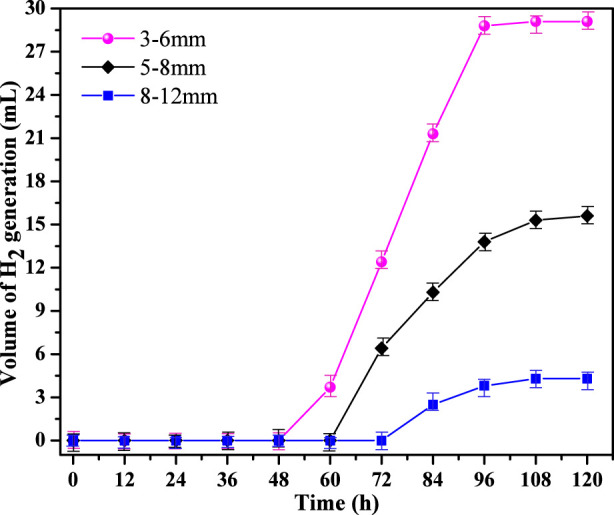
Effect of Fe-C filler particle size on H_2_ generation in the reaction system.

One of the most distinctive technical features of this process is the in-situ generation of hydrogen synchronously during wastewater treatment. As illustrated in Figure 6, gas evolution was detected in all three groups of Fe-C micro-electrolysis fillers with different particle sizes during the reaction. Moreover, the cumulative hydrogen collection volume increased significantly with the extension of reaction time and the decrease in filler particle size, reaching 291 mL, 154 mL, and 43 mL, respectively (based on 1.0 L of acidified oil wastewater). Notably, no gas generation was observed within the initial 48 h of the reaction. A reasonable inference is that hydrogen ions in the system preferentially participate in reduction reactions in the form of adsorbed hydrogen atoms. Specifically, the hydrogen atoms adsorbed on the cathode surface reduce nitrogen-containing oxidized pollutants (e.g., nitrate and nitrite) to amino groups (Cai et al., 2024), and this process is further corroborated by the significant increase in NH_4_^+^-N concentration within the initial 0–12 h, as shown in Figure 5b. Additionally, as illustrated in Figure 3, the Fe^2+^ release rate was the highest within the initial 48 h, indicating that the Fe-C micro-electrolysis reaction was intense. A large number of electrons were transferred to the cathode, where H^+^ was reduced to adsorbed hydrogen atoms, which were utilized for the reduction of organic pollutants and nitrogen-containing compounds rather than the formation of free H_2_.

In the Fe-C micro-electrolysis system, the electrochemical reaction (2H^+^ + 2e^−^ → H_2_) occurring on the surface of the cathode (carbon phase) not only effectively increased the wastewater pH but also realized in-situ hydrogen production. It is thus evident that Fe-C micro-electrolysis is not only an efficient wastewater purification technology but also possesses potential energy recovery functionality. The generation mechanism of hydrogen originates from the electrochemical reduction of protons at the cathodic sites on the carbon material surface (Pan et al., 2025). Given that the wastewater was strongly acidic (pH < 3), the system exhibited excellent thermodynamic driving force and fast kinetic rate for hydrogen evolution at the initial reaction stage. Traditional viewpoints often regard high acidity as an adverse factor for the operation of micro-electrolysis processes (Ahmed et al., 2024). However, from the perspective of hydrogen energy production, the acidic environment precisely constitutes a key operational advantage for the spontaneous hydrogen evolution of this process. Although the total amount of hydrogen obtained in the batch experiment was limited, this finding holds significant theoretical value: it confirms that the chemical potential energy contained in acidic industrial wastewater can be partially converted into usable clean energy carriers (H_2_) through the Fe-C micro-electrolysis process, thereby avoiding the dependence on alkaline neutralizing agents.

### 3.6 Mineralogical characteristics of the recovered precipitate

**FIGURE 7:**
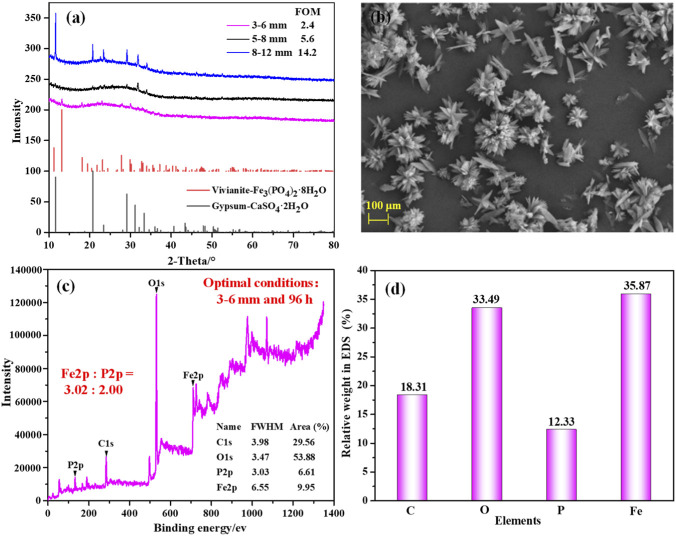
Characterization and analysis of the recovered precipitate were performed by XRD **(a)**, SEM **(b)**, XPS **(c)**, and EDS **(d)**.

Whether the solid product in this process is successfully converted into economically valuable vivianite is a critical factor determining the process feasibility. It should be emphasized that merely achieving phosphorus removal is not equivalent to realizing selective resource recovery of phosphorus. In most iron-based phosphorus removal systems, phosphorus often exists in the form of amorphous iron hydroxide/iron phosphate coprecipitates or sludge-like substances (Guan et al., 2021), whose mineralogical properties are difficult to define, limiting their subsequent resource utilization. Systematic characterizations in this study confirmed that well-crystallized vivianite could be efficiently generated *in situ* when Fe-C fillers with particle sizes of 3–6 mm and 5–8 mm were adopted (Figure 7). Specifically, the product obtained with 3–6 mm fillers exhibited a lower figure of merit (FOM) of 2.4, along with sharper and more symmetric XRD characteristic diffraction peaks, indicating superior crystal integrity and crystallinity (Figure 7a). In contrast, the product obtained with 8–12 mm fillers was identified as CaSO_4_.2H_2_O by XRD analysis. This phenomenon is inferred to be attributed to the increased mass transfer resistance and limited reaction kinetics caused by the larger particle size, which prevented the system pH and dissolved Fe^2+^ concentration from reaching the thermodynamic thresholds required for vivianite crystallization (pH ≈ 4.5–7.0, Fe^2+^ > 10^−4^ moL/L) (Liu et al., 2018; Paskin et al., 2025). Notably, high concentrations of Ca^2+^ and SO_4_^2-^ coexisted in the reaction solution; under strongly acidic conditions (pH < 3.5, Supplementary Figure S1), the ion product of CaSO_4_.2H_2_O first reached supersaturation, leading to its preferential precipitation and thus inhibiting vivianite nucleation.

In Figure 7b, SEM observations further revealed that the recovered solid exhibited a typical petal-like and plate-like laminated structure, which was highly consistent with the vivianite crystal growth morphology reported in the literature (Karimian et al., 2026). This structural evolution conforms to the typical anisotropic growth mechanism of vivianite (i.e., gradual development from initial plate-like nuclei to three-dimensional petal-like aggregates) (Ogorodova et al., 2017). The volume-averaged particle size (d_50_) of the product was measured to be 138.7 μm, indicating sufficient crystal growth. Large-sized crystals have dual significance for improving product purity and solid-liquid separation efficiency.

Additionally, XPS analysis (Figure 7c) further verified the elemental composition and chemical state of the product: clear characteristic peaks were observed at 712.6 eV (Fe2p), 531.8 eV (O1s), 287.5 eV (C1s), and 133.6 eV (P2p), confirming the coexistence of Fe, O, P, and C elements. Critically, the peak area ratio of Fe 2p to P 2p was 3.02:2.00 (Supplementary Figure S2), which was highly consistent with the theoretical atomic ratio (3:2) of the ideal vivianite formula (Fe_3_(PO_4_)_2_·8H_2_O) (Hao et al., 2018). EDS mapping results (Figure 7d) showed uniform distribution of C, Fe, P, and O elements. Quantitative analysis indicated that the mass fractions of Fe and P on the crystal surface were 35.87% and 12.33%, respectively, with a deviation of less than ±3.5% from the theoretical values (Fe: 34.58%, P: 12.76%) (Moulessehoul et al., 2024), further confirming the high purity of the product.

Finally, under the optimal operating parameters of the Fe-C micro-electrolysis method for treating acidified oil wastewater (electrolysis time of 96 h and filler particle size of 3–6 mm), it was achieved that 100 ± 5 g of vivianite could be produced per liter of wastewater treated. This process demonstrated that the use of Fe-C filler can effectively achieve in-situ, highly selective, and high-purity vivianite crystallization and recovery without the addition of external iron salts or alkalis, exhibiting significant resource utilization potential.

### 3.7 Significance of this study and future perspectives

In this study, the Fe-C micro-electrolysis system realizes the continuous and controllable in-situ release of Fe^2+^ through the electrochemical corrosion mechanism, and spontaneously consumes H^+^ via the cathodic hydrogen evolution reaction, driving the system pH to gradually increase from the initial acidic range (pH = 1–2) to the stable crystallization interval of vivianite (pH = 5.5–7.0). Thus, an integrated “reaction-iron release-pH adjustment-crystallization” process is constructed. The core advantages of this strategy are reflected in the following five aspects: (i) No additional alkali needs to be added to the reaction system. Instead, the system achieves an elevation of pH (reaching 5.6) through cathodic reduction reactions; (ii) No exogenous iron salt addition. Fe^2+^ is continuously supplied by the anodic oxidation of Fe^0^ in the fillers, which possesses both reaction activity and material cycling properties; (iii) Synchronous hydrogen production. Cathodic hydrogen evolution not only regulates pH but also converts part of the protons in the wastewater into high-grade clean energy (H_2_), endowing the process with energy recovery potential; (iv) Synergistic pollutant reduction. This work innovatively achieves the synergistic integration of phosphorus recovery, in-situ hydrogen production, and multi-pollutant (COD and NH_4_^+^-N) removal in a single system, whereas previous studies primarily focused on single phosphorus recovery without considering energy recovery or comprehensive pollutant treatment; (v) Potential for engineering application. The use of commercial Fe-C fillers makes the process highly attractive for engineering implementation, and no complex chemical dosing system is required, which significantly reduces the thresholds of infrastructure construction and operation and maintenance.

Although this study has achieved significant progress in alkali-free phosphorus recovery and multi-functional synergy from acidified oil wastewater, there are still some limitations that need to be addressed in future research to promote the industrial application and performance upgrading of the process.

- The crystallization kinetics of vivianite in the batch Fe-C micro-electrolysis system is relatively slow, resulting in a long overall reaction cycle (120 h), which limits the treatment efficiency in industrial applications. Future research should focus on optimizing the Fe-C filler structure to accelerate the Fe^0^ corrosion rate and Fe^2+^ release flux. For example, introducing catalytic additives (e.g., transition metals or metal oxides) to modify the Fe-C interface, or optimizing the pore structure of fillers to increase the density of electrochemically active sites. In addition, the introduction of vivianite seed crystals or the regulation of interfacial nucleation conditions can effectively shorten the crystallization induction period and improve the crystallization rate, thereby reducing the reaction cycle.
- The current phosphorus recovery rate (84.7%) of the process is 5%–10% lower than that of optimized traditional chemical crystallization methods, and the purity of vivianite may be affected by the co-precipitation of impurities such as CaSO_4_.2H_2_O in practical application. Future work should explore the regulation strategies of impurity inhibition. For example, pre-removing SO_4_^2-^ or Ca^2+^ in wastewater through pre-treatment processes, or optimizing the reaction conditions to enhance the selective crystallization of vivianite. Meanwhile, the optimization of Fe-C filler particle size gradation can further improve the mass transfer efficiency and reaction uniformity, thereby improving the phosphorus recovery rate and product purity.
- The long-term operational stability of Fe-C fillers is a key factor affecting the industrial application of the process. During the reaction, the formation of Fe^3+^/CaSO_4_ covering layers on the filler surface will lead to filler passivation, reducing the reaction activity and service life. The Fe-C filler can maintain excellent electrochemical activity and reaction efficiency for 10 batch reactions (each batch lasting 96 h) under the experimental conditions of this study, before obvious passivation or activity attenuation occurs. Thus, future work should focus on the following two aspects: (1) developing anti-passivation modification technologies for Fe-C fillers (e.g., surface coating, composite filler preparation) to extend their service life; (2) exploring efficient regeneration methods for spent Fe-C fillers to reduce the overall process cost and improve resource utilization efficiency.
- The current study is based on batch experiments, and the performance of the process in continuous flow reactors needs to be verified. Future research should design and optimize the continuous flow reactor configuration, strengthen the gas-liquid-solid separation system and online hydrogen collection technology, and solve the problems of filler loss, hydrogen escape, and crystal deposition in continuous operation. Meanwhile, pilot-scale experiments should be carried out to evaluate the process performance under actual wastewater conditions, providing data support for industrial amplification.
- The coupled integration of the Fe-C micro-electrolysis process with other technologies can further expand its application scope and comprehensive benefits. For example, coupling with fuel cells or hydrogen storage modules can realize the efficient utilization of in-situ generated hydrogen; coupling with biological treatment processes can further improve the removal efficiency of refractory organic pollutants, realizing the deep purification of wastewater. In addition, the life cycle assessment of the process should be carried out to comprehensively evaluate its environmental impact and economic feasibility, providing a scientific basis for its industrial popularization and application.

## 4 Conclusion

In this study, an Fe-C micro-electrolysis process was successfully developed for phosphorus recovery from acidified oil wastewater via in-situ vivianite crystallization. The results demonstrated that commercial Fe-C fillers could release Fe^2+^ through sacrificial corrosion and consume protons via cathodic reactions, thereby creating favorable conditions for vivianite formation without the exogenous addition of iron salts or alkaline reagents. The Fe-C fillers with a particle size of 3–6 mm exhibited the optimal overall performance: after 96 h of reaction, the wastewater pH was elevated to 5.6, with the phosphorus recovery efficiency reaching 84.7% and the vivianite crystallization rate attaining 82.3%. Concurrently, per liter of wastewater treated, the process yielded 291 mL of H_2_ and approximately 100 g of vivianite, while simultaneously removing 23.2% of NH_4_^+^-N and 48.2% of COD. Compared with traditional vivianite crystallization methods, the Fe-C micro-electrolysis process has significant advantages, namely, extremely low chemical reagent consumption in the process, simultaneous hydrogen production, and synergistic removal of multiple pollutants in wastewater. Overall, this study provides a feasible and promising strategy for the sustainable treatment and resource recovery of acidic phosphorus-rich industrial wastewater.

## Data Availability

The original contributions presented in the study are included in the article/Supplementary Material, further inquiries can be directed to the corresponding authors.
