# Supplementary material for: Simultaneous phosphorus recovery as vivianite crystallization and hydrogen generation from acidified oil wastewater by Fe-C micro-electrolysis
Source: Front Chem. 2026 Jun 15;14:1866760. doi: 10.3389/fchem.2026.1866760 (PMC13310974; doi:10.3389/fchem.2026.1866760)
Supplement: Supplementary file 1 [file DataSheet1.docx]

**Supporting Information**

***Simultaneous phosphorus recovery as vivianite*** ***crystallization and hydrogen generation from acidified oil wastewater by Fe-C micro-electrolysis***

Kai Cui ^a^*, Guangyu Xu ^a^, Hong Zhang ^a^, Jinpeng Yu ^a,b^, Fei Ma ^a^, Kun Guo ^a^*

^a^ *School of Chemical Engineering and Technology, Xi’an Jiaotong University, Xi’an 710049, China*

^b^ *Shenmu Fuyou Energy Technology Co., Ltd, Shenmu, Shanxi 719319, China*

***** Corresponding Author:

Kai Cui, Email: cuikai212@xjtu.edu.cn

Kun Guo, Email: [kun.guo@xjtu.edu.cn](mailto:kun.guo@xjtu.edu.cn)

**Figure S1.** The concentrations of Fe^2+^ and PO_4_^3-^ in the solution could satisfy the threshold of the thermodynamic saturation index (SI) for vivianite precipitation.

**Figure S2.** XPS pattern and surface morphology of the recovered precipitation.

**Table S1.** The Hupfer extraction method.

| Phosphorus  components | Extractant | Concentration  (mol/L) | Extraction duration (min) | Solid-to-liquid Ration  (mL: gDS) |
| --- | --- | --- | --- | --- |
| Labile-P  MCO_3_-P  (Fe-P+Al-P+Org-P) ^a^  Ca-P  Residual-P | deionized water  acetic acid  NaOH  HCl  HNO_3_ | -  0.10  1.00  0.50  14.4 | 20  80  1080  1080  60 | 60:1  60:1  60:1  60:1  60:1 |

^a^ The total is regarded as Fe-P because the raw wastewater does not contain Al^3+^ or organic matter.

During the Hupfer fractionation procedure, phosphorus in the precipitates was predominantly classified into five operationally defined fractions: (1) labile water-soluble phosphorus (Labile-P), extracted with deionized water; (2) carbonate-bound phosphorus (MCO₃-P), extracted using 0.1 mol/L acetic acid solution; (3) iron-bound phosphorus (Fe-P, the dominant fraction for vivianite), aluminum-bound phosphorus (Al-P), and organic phosphorus (Org-P), extracted with 1 mol/L sodium hydroxide solution; (4) calcium-bound phosphorus (Ca-P), extracted via 0.5 mol/L hydrochloric acid solution; and (5) residual phosphorus (Residual-P), obtained by digestion with concentrated nitric acid.
